# NUTRIC Score Is Not Superior to mNUTRIC Score in Prediction of Mortality of COVID-19 Patients

**DOI:** 10.1155/2022/1864776

**Published:** 2022-01-31

**Authors:** Berkay Kucuk, Sevil Baltaci Ozen, Gul Meral Kocabeyoglu, Nevzat Mehmet Mutlu, Esra Cakir, Isil Ozkocak Turan

**Affiliations:** ^1^Department of Critical Care, Hatay Education and Research Hospital, Hatay, Turkey; ^2^Department of Critical Care, Yenimahalle Education and Research Hospital, Ankara, Turkey; ^3^Department of Critical Care, Ankara City Hospital, Ankara, Turkey

## Abstract

**Objectives:**

The NUTRIC (nutrition risk in the critically ill) score and the modified NUTRIC score are two scoring systems that show the nutritional risk status and severity of acute disease of patients. The only difference between them is the examination of interleukin-6 (IL-6) level. The aim of this study was to investigate whether or not the NUTRIC score is superior to the mNUTRIC score in the prediction of mortality of patients with COVID-19 followed up in the Intensive Care Unit (ICU). *Material and Method*. This retrospective study included 322 patients followed up in ICU with a diagnosis of COVID-19. A record was made of demographic data, laboratory values, clinical results, and mortality status. All the data of the patients were compared between high and low variations of the NUTRIC score and the mNUTRIC score.

**Results:**

A high NUTRIC score was determined in 62 patients and a high mNUTRIC score in 86 patients. The need for invasive mechanical ventilation, the use of vasopressors in ICU, the development of acute kidney injury, and mortality rates were statistically significantly higher in the patients with high NUTRIC and high mNUTRIC scores than in those with low scores (*p* = 0.0001 for all). The AUC values were 0.791 for high NUTRIC score and 0.786 for high mNUTRIC score (*p* = 0.0001 for both). No statistically significant difference was determined between the two scoring systems.

**Conclusion:**

Although the NUTRIC score was seen to be superior to the mNUTRIC score, no statistically significant difference was determined. Therefore, when IL-6 cannot be examined, the mNUTRIC score can be considered safe and effective for the prediction of mortality in COVID-19 patients.

## 1. Introduction

At the end of December 2019, a new coronavirus (2019-nCoV, SARS-CoV-2) was identified in a case series of pneumonia with rapid person-to-person infection in the city of Wuhan, Hubei Province, China [1]. In February 2020, this pneumonia was named coronavirus disease 2019 (COVID-19) by the World Health Organization (WHO) [2]. The disease has spread rapidly all over the world from that date on. The WHO declared a pandemic on March 11^th^, 2020. On the same day, the first case of COVID-19 was recorded in Turkey [3]. As there is no specific antiviral treatment, there continue to be hundreds of thousands of cases determined throughout the world each day and thousands of deaths. As of May 2021, case numbers have exceeded 170 million worldwide, and the number of deaths due to COVID-19 is more than 3.5 million [4].

The virus is spread through aerosol droplets in COVID-19 infection and most patients can have a mild disease course with symptoms such as fever, listlessness, muscle and joint pains, loss of appetite, headache, nausea, vomiting, loss of taste and smell, and cough [5]. In more severe cases, pneumonia, acute respiratory distress syndrome (ARDS), respiratory failure, renal failure, sepsis, septic shock, multiple organ failure, and even death may be seen. The Centre for Disease Control (CDC) has defined COVID-19 with a broad disease spectrum from mild disease to critical disease. Mild disease is described as uncomplicated upper respiratory tract viral infection and moderate disease as pneumonia which does not require oxygen support [6]. These two patient groups are treated and followed up at home and in hospital wards. However, severe pneumonia includes pneumonia accompanied by dyspnea, respiratory problems, central oxygen saturation (SpO_2_) ≤93%, and a ratio of partial arterial oxygen pressure to fractioned oxygen saturation (pO_2_/FiO_2_) of <300, and critical disease is defined as respiratory failure, septic shock, and multiple organ dysfunction/failure [6]. These two patient groups are followed up in the Intensive Care Unit (ICU). Although this classification provides important guidance about disease prognosis and mortality, the length of hospital stay of critical patients and those with severe pneumonia increases morbidity, mortality, and hospital costs [7].

There is still no appropriate method or clinical scoring system to show the severity of COVID-19 and predict disease course. To date, several scoring systems have been used to predict the severity of COVID-19 and show prognosis, including the Acute Physiology and Chronic Health Evaluation II (APACHE II), Sequential Organ Function Assessment (SOFA), Pneumonia Severity Index (PSI), Combination of Confusion, Urea, Respiratory Rate, Blood Pressure, and Age ≥65 (CURB-65), and Modified Early Warning Score (MEWS) [8, 9]. In addition, many data such as sodium changes and lipid metabolism have been used to understand the pathophysiological changes in COVID-19 [10, 11]. Some nomograms have been developed to examine the severity and progression of the disease [12].

Heyland et al. developed the Nutrition Risk in the Critically Ill (NUTRIC) score as a scoring system specific to ICU patients for the evaluation of the nutritional risk of patients. This scoring system is formed from age, APACHE II score, SOFA score, the number of comorbidities, the length of hospital stay before admittance to ICU, and serum interleukin-6 (IL-6) level. The NUTRIC score not only evaluates the nutritional status of the patient but also helps to determine the severity of acute disease [13]. Patients are separated into low-risk (0–5) and high-risk (6–10) groups according to the NUTRIC score. Patients with a high NUTRIC score require more active and effective nutritional support and are associated with more negative clinical outcomes [13]. In another version of the NUTRIC score, Rahman et al. removed the serum IL-6 level but kept the other components and named this the modified NUTRIC (mNUTRIC) score [14].

There is extremely limited information related to the nutritional risk status, clinical outcomes, and disease course of COVID-19 patients followed up in ICU. Different studies have used the NUTRIC score and the mNUTRIC score to evaluate the nutritional status of COVID-19 patients, but no study could be found in the literature that has compared these two scores.

Therefore, the aim of this retrospective study was to compare the efficacy of the NUTRIC score and the mNUTRIC score in the evaluation of the nutrition risk and the prediction of the clinical outcomes and mortality in COVID-19 patients followed up in ICU.

## 2. Materials and Methods

### 2.1. Ethical Considerations

Approval for this retrospective study was granted by the Clinical Research Ethics Committee of Ankara City Hospital No. 1 (decision no. E1-20-134, date: December 9, 2020.

### 2.2. Study Population and Protocol

This retrospective study included the data of patients aged 18 years who were followed up in three COVID-19 ICUs in Ankara City Hospital between June 1^st^ and November 1^st^, 2020. The diagnosis of COVID-19 was made from a positive reverse transcriptase polymerase chain reaction test (RT-PCR) and/or thorax computed tomography (CT) findings consistent with COVID-19. The patients followed up in ICU were severe pneumonia cases and critical patients.

The nutritional requirements and support of the patients were planned with the general consensus of the ICU team. For those who could not have oral intake with no contraindication for enteral nutrition, a nasogastric tube was attached within the first 24 hours and enteral nutrition was started. For patients who could not receive enteral nutrition, parenteral nutrition support was applied.

Patients were excluded from the study if they remained in ICU for less than 24 hours or if the medical data were incomplete.

### 2.3. Data Collection

Data of the patients were retrieved from the hospital information management system and the patient observation forms in respect of age, gender, history of chronic diseases, comorbidities, laboratory values, length of stay in ICU, length of stay in hospital before admittance to ICU, total length of stay in hospital, disease outcome (survival/exitus), disease severity (severe pneumonia/critical disease), Glasgow Coma Score (GCS), noninvasive oxygen treatments (nasal mask, nasal high-flow, and noninvasive mechanical ventilation), the requirement for invasive mechanical ventilation, the duration of invasive mechanical ventilation, the nutritional route (enteral, parenteral, and oral), the requirement for vasopressors, development of acute kidney injury, and the requirement for renal replacement therapy.

Evaluation of acute kidney injury was made using the KDIGO classification (Kidney Disease: Improving Global Outcomes) [15]. Evaluations of arterial blood gas and biochemical parameters were made using the blood samples taken on admittance to ICU. Complete blood count (neutrophil, lymphocyte, haemoglobin, platelet, and white blood cell), neutrophil/lymphocyte ratio (NLR), creatinine, total bilirubin, albumin, procalcitonin, C-reactive protein (CRP), IL-6, ferritin, D-dimer, and lactate values were obtained from the hospital information system. The APACHE II and SOFA scores calculated within the first 24 hours of admittance to ICU were recorded. The NUTRIC score (0–10) and the mNUTRIC score (0–9) were calculated and recorded. A NUTRIC score of ≥6 and mNUTRIC score of ≥5 were accepted as high risk. The components and scoring of the NUTRIC and mNUTRIC scores are shown in Table 1.

### 2.4. Statistical Analysis

Data obtained in the study were analyzed statistically using SPSS vn. 25.0 software. Categorical variables were stated as number and percentage and continuous variables as mean ± standard deviation (SD) values, or if not conforming to normal distribution, as median, minimum, and maximum values. In the comparisons of categorical variables, the chi-square test or the Fisher test was used. In the comparisons of continuous measurements between groups, according to the number of variables, the Student's *t*-test was used for data showing normal distribution and the Mann–Whitney *U* test for data not conforming to normal distribution. In the determination of cutoff values for the scoring systems used in the study, sensitivity and specificity values were calculated and the area under the ROC curve (AUC) was evaluated with ROC analysis. In all the tests, a value of *p* < 0.05 was accepted as the level of statistical significance.

## 3. Results

Evaluation was made of a total of 322 COVID-19 patients, comprising 62.1% males and 37.9% females with a mean age of 69.1 ± 14.4 years (interquartile range (IQR): 19–99 years). No comorbidities were determined in 15.2% of the patients. The most common comorbidities were hypertension (63%), diabetes mellitus (39.4%), and coronary artery disease (29.2%). According to the CDC criteria, the majority of the patients (79.2%) were admitted to ICU with severe pneumonia and the remainder (20.8%) were critical patients. Mortality developed in 57.5% of the patients.

The median APACHE II score was 11 (IQR: 2–39), the median SOFA score was 3 (IQR: 1–16), and the median Glasgow Coma Score was 15 (IQR: 3–15). A total of 189 (58.7%) patients required invasive mechanical ventilation and vasopressor drugs were required by 145 (45%) patients. Acute kidney injury developed in 105 (32.6%) patients, and intermittent dialysis or continuous renal replacement therapy (CRRT) was applied to 71 (22%). The median length of stay in ICU and total length of stay in hospital were 9 days (IQR: 2–43) and 14 days (IQR: 2–69), respectively. The demographic and clinical data and the laboratory test results of the patients are shown in Tables 2 and 3.

A high NUTRIC score (≥6) was determined in 62 patients and a high mNUTRIC score (≥5) in 86 patients. The median age was determined to be statistically significantly older in the patients with a high NUTRIC score compared to those with a low score (*p* = 0.0001). In patients with a high NUTRIC score, heart failure, chronic renal failure, and a history of cerebrovascular event were determined more than in patients with a low score (*p* = 0.029, *p* = 0.0001, and *p* = 0.024). The length of stay in ICU and total length of stay in hospital were determined to be significantly shorter in those with a high NUTRIC score compared to those with a low score (*p* = 0.036 and *p* = 0.001, respectively). The GCS of patients with a high NUTRIC score were determined to be significantly lower than those of the patients with low NUTRIC scores (*p* = 0.0001).

Statistically significantly higher median APACHE II and SOFA scores were determined in patients with high NUTRIC scores compared to those with low scores (*p* = 0.0001 and *p* = 0.0001, respectively). Critical disease was seen more together with a high NUTRIC score compared to those with a low NUTRIC score (*p* = 0.0001). The requirement for invasive mechanical ventilation, requirement for vasopressors on admittance to ICU, the use of vasopressors in ICU, development of acute kidney injury, the need for dialysis or CRRT, and mortality rates were determined to be higher in those with a high NUTRIC score compared to those with a low score (*p* = 0.0001 for all).

In the laboratory test results, levels of creatinine, total bilirubin, procalcitonin, ferritin, WBC, NLR, lactate, and IL-6 were determined to be significantly higher (*p* = 0.0001, *p* = 0.029, *p* = 0.001, *p* = 0.0001, *p* = 0.0001, *p* = 0.01, *p* = 0.031, and *p* = 0.0001, respectively), and albumin, lymphocyte, and haemoglobin values were significantly lower (*p* = 0.0001, *p* = 0.031, and *p* = 0.0001, respectively) in patients with a high NUTRIC score.

In the patients with a high mNUTRIC score, age was determined to be statistically significantly older compared to those with a low score (*p* = 0.0001). In patients with a high mNUTRIC score, heart failure, arrhythmia, chronic renal failure, history of cerebrovascular event, and Alzheimer's disease were determined more than in patients with a low score (*p* = 0.014, *p* = 0.01, *p* = 0.0001, *p* = 0.024, and *p* = 0.009, respectively). The total length of stay in hospital was determined to be significantly shorter in those with a high mNUTRIC score compared to those with a low score (*p* = 0.001). The GCS of patients with a high mNUTRIC score were determined to be significantly lower (*p* = 0.0001).

Statistically significantly higher median APACHE II and SOFA scores were determined in patients with high mNUTRIC scores compared to those with low scores (*p* = 0.0001 and *p* = 0.0001, respectively). Critical disease was seen more in patients with a high mNUTRIC score than in those with a low mNUTRIC score (*p* = 0.0001). The requirement for invasive mechanical ventilation, requirement for vasopressors on admittance to ICU, the use of vasopressors in ICU, development of acute kidney injury, the need for dialysis or CRRT, and mortality rates were determined to be higher in those with a high mNUTRIC score compared to those with a low score (*p* = 0.0001 for all).

In the laboratory test results, levels of creatinine, D-dimer, procalcitonin, ferritin, WBC, neutrophils, NLR, lactate, and IL-6 were determined to be significantly higher (*p* = 0.0001, *p* = 0.027, *p* = 0.009, *p* = 0.0001, *p* = 0.0001, *p* = 0.016, *p* = 0.023, and *p* = 0.001, respectively), and albumin, haemoglobin, and thrombocyte values were significantly lower (*p* = 0.0001, *p* = 0.0001, and *p* = 0.041, respectively) in patients with a high mNUTRIC score. The comparisons of the NUTRIC scores and mNUTRIC scores are shown in Table 4.

**Figure 1:**
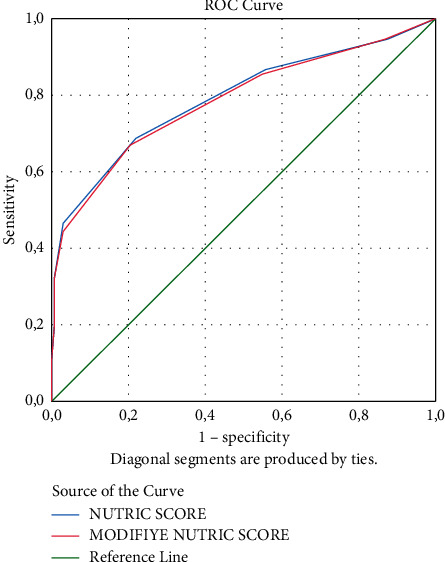
Performance of NUTRIC score and modified NUTRIC score in predicting mortality.

**Figure 2:**
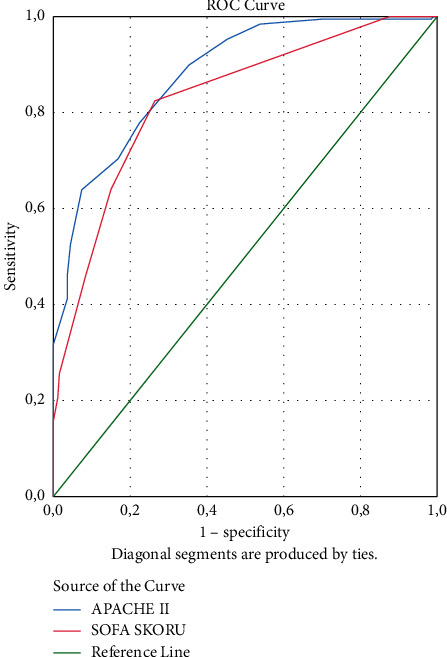
Performance of APACHE II score and SOFA score in predicting mortality.

The area under the curve (AUC) values were found to be 0.791 (95% CI: 0.743–0.840) for the NUTRIC score and 0.786 (95% CI: 0.737–0.835) for the mNUTRIC score for the prediction of mortality, with no statistically significant difference (Figure 1). In the ROC curve analysis of the NUTRIC score, the best cutoff value was 3.5, with sensitivity of 68.6% and specificity of 79.1%. For the mNUTRIC score, the best cutoff value on the ROC curve was 3.5 with sensitivity of 67.7% and specificity of 80.0%. The AUC values of the scores are shown in Table 5. The AUCs of APACHE II score and SOFA score were 0.880 (95% CI: 0.845–0.916) and 0.829 (95% CI: 0.785–0.874), respectively (Figure 2).

## 4. Discussion

COVID-19 disease, which caused the death of millions of people, has become a destructive pandemic [1, 2]. As there is still no safe and effective treatment for COVID-19, which can lead to a severe clinical status, many studies continue to be conducted on the risk factors of the disease, clinical outcomes, disease course, morbidity, and mortality. Especially in patients with critical disease followed up in ICU, the nutritional risk status affects clinical outcomes [16].

The NUTRIC score, which was developed by Heyland et al. and later modified by Rahman et al., not only evaluates the nutritional status of patients but also helps in determining the severity of acute disease [13, 14]. Although there are many studies in literature related to the both scores, to the best of our knowledge, there has been no previous study comparing the both scores in COVID-19 patients followed up in ICU.

Values of ≥6 for the NUTRIC score and ≥5 for the mNUTRIC score have been defined as high scores. Just as patients with high scores require more active and effective nutritional support, they are also associated with more negative clinical outcomes [13, 14]. Of the total 322 patients included in this study, 62 had a high NUTRIC score and 86 had a high mNUTRIC score. The patients with high scores were older (*p* = 0.0001), the length of stay in ICU and total hospital stays were shorter, and the APACHE II and SOFA scores were determined to be higher compared to patients with low scores (*p* = 0.0001). It has been reported that according to the CDC criteria, 80% of COVID-19 patients are asymptomatic, 15% have severe pneumonia, and 5% are critical patients [6]. Asymptomatic patients and those with mild disease are followed up at home or in hospital wards.

The patients followed up in ICU in the current study were those with severe pneumonia or critical patients. The rate of critical disease seen in patients with high NUTRIC and mNUTRIC scores was significantly greater (*p* = 0.0001). The mortality rates of patients, the requirement for invasive mechanical ventilation, the use of vasopressors on admittance to ICU, the use of vasopressors while in ICU, the development of acute kidney injury, and the requirement for dialysis or CRRT were significantly higher in patients with high NUTRIC and mNUTRIC scores compared to those with low scores (*p* = 0.0001). The IL-6 levels were also found to be significantly higher in patients with high NUTRIC and mNUTRIC scores.

In a study by Zhang et al. of 136 COVID-19 patients, those with a high mNUTRIC score were compared with those with a low score, and similar to the current study, a statistically significant difference was reported in respect of age, APACHE II score, SOFA score, GCS, the use of vasopressors, and mortality (*p* < 0.001 for all) [17]. Li et al. evaluated 211 COVID-19 patients followed up in ICU and also reported a high rate of in-hospital mortality for patients with a high mNUTRIC score (*p* < 0.001) [18]. In a study by Osuna-Padilla et al. of 112 COVID-19 patients who required mechanical ventilation, those with a high mNUTRIC score were determined to have high mortality rates (*p* = 0.03) [19]. In these other studies, only patients with high and low mNUTRIC scores have been evaluated, whereas the current study has the distinguishing feature of being the first study in literature to have evaluated both the NUTRIC score and the mNUTRIC score in COVID-19 patients.

Jeong et al. examined both the NUTRIC and mNUTRIC scores in sepsis patients and reported that advanced age, APACHE II score, SOFA score, the use of vasopressors, the need for CRRT, and mortality rates were statistically significantly different in patients with high NUTRIC and mNUTRIC scores compared to those with low scores, and the total length of stay in hospital was longer (*p* < 0.001) [20]. Those findings are similar to the current study results, with the exception that the length of stay in ICU was longer in the patients with low NUTRIC and mNUTRIC scores (*p* = 0.036 and *p* = 0.142). This could have been due to there being no proven treatment for COVID-19 and that the disease course led more rapidly to mortality in critical patients with severe pneumonia or who developed organ failure. The COVID-19 pandemic has affected the whole healthcare system and so for associated reasons such as delays in transferring patients to ICU, shortcomings in the availability of invasive or noninvasive oxygen support systems, and insufficient numbers of healthcare personnel, which shortened the time to mortality, there may have been a shorter length of stay in ICU. In addition, the longer period of hypoxemia and the need for oxygen of patients with low scores and delays in discharge from ICU because of following the defined quarantine periods could have prolonged the length of stay in ICU of patients with low scores. In a study by Mayr et al., 114 patients with end-stage liver disease were evaluated in respect of NUTRIC and mNUTRIC scores. Age, IL-6, APACHE II score, SOFA score, and mortality rates were determined to be higher in patients with high NUTRIC and mNUTRIC scores compared to those with low scores (*p* < 0.001) [21]. The findings of that study were similar to the current study results.

The AUCs of the NUTRIC and mNUTRIC scores predicting mortality were 0.791 and 0.786, respectively, with no significant difference determined between the two scores. The AUCs of the APACHE II score and SOFA score were 0.880 and 0.829, respectively. In the ROC curve of the NUTRIC and mNUTRIC scores, the best cutoff value was 3.5.

In the study by Mayr et al. of patients with end-stage liver disease, the AUC for prognostic value of the NUTRIC score was 0.806, and for the mNUTRIC score, 0.788. The highest combined sensitivity and specificity was found with a cutoff value of ≥7. Both the scores were determined to perform better than APACHE II (AUC = 0.745) and SOFA (AUC = 0.778) [21]. In the current study, the APACHE II and SOFA scores were higher. This situation of patients with high APACHE II and SOFA scores could have been the result of parameters related to various organ systems being affected because of patients in the current study with multiple organ failure, deep hypoxia, and comorbidities. The low APACHE II and SOFA scores of patients admitted to ICU with severe hypoxemia who did not develop other organ failure, did not have a decrease in GCS, did not use vasopressors, and did not develop renal failure were considered to contribute to this result.

Jeong et al. examined 482 sepsis patients and reported AUC of 0.762 (95% CI: 0.718–0.806) for the NUTRIC score and 0.757 (95% CI: 0.713–0.801) for the mNUTRIC score in showing 28-day mortality, with no statistically significant difference (*p* = 0.45). The best cutoff value of the ROC curve for the mNUTRIC score was 6 with sensitivity of 75% and specificity of 65% [20]. In another study by de Vries et al. of patients followed up on mechanical ventilation, the AUC was reported to be 0.768 (95% CI: 0.722–0.814) for the mNUTRIC score in showing mortality and the cutoff value was found to be > 4 [22]. Brascher et al., on the other hand, determined the NUTRIC score to be AUC of 0.79 (95% CI: 0.67–0.89) and the best cutoff value of 5 (64.7% sensitivity and 66.7% specificity) to indicate mortality [23]. The AUC values in the current study were consistent with the finding of 0.783 reported in the first study by Heyland et al. [13].

IL-6 is an important marker of inflammation, and just as its relationship with many other diseases has been investigated, so there are also many recent studies showing an association with COVID-19 [24–26]. Those studies have shown that high IL-6 levels are associated with poor prognosis and mortality, and there was a similar correlation in the current study in patients with high NUTRIC and mNUTRIC scores [27–29]. The current study is the first in literature to have compared the NUTRIC score and the mNUTRIC score in COVID-19 patients followed up in ICU. The only difference between the two scoring systems is the inclusion of IL-6 in the score calculations. As additional costs are incurred, IL-6 is not routinely examined in all ICUs. In the current study, although the NUTRIC score was superior to the mNUTRIC score in the prediction of mortality, the difference was not statistically significant. Therefore, routine evaluation of IL-6 may not be necessary, which may therefore be more convenient in both economic terms and in respect of score calculation. Thus, the mNUTRIC score can be as safely used in COVID-19 patients as the NUTRIC score.

## 5. Limitations

As this was a retrospective study, the potential effect of the medical condition on height, weight, and body mass index was not taken into consideration from the medical records. As the records may have not been complete during the intense period of the pandemic, the effects of nutritional support on clinical course and mortality could not be clearly evaluated.

## 6. Conclusions

The results of this study demonstrated that after the APACHE II and SOFA scores, the NUTRIC and mNUTRIC scores were effective scoring systems in COVID-19 patients in ICU, and due to the lower cost and ease of calculation of the mNUTRIC score, it could be considered in preference to the NUTRIC score.

## Figures and Tables

**Table 1:** Classifications of the NUTRIC score and the modified NUTRIC score.

Components	Scoring system			
	0	1	2	3
Age (years)	<50	50–75	≥75	
APACHE II	<15	15–20	20–28	≥28
SOFA	6	6–10	≥10	
Number of comorbidities	0–1	≥2		
Length of stay in hospital before admittance to ICU	<1 day	≥1 day		
Interleukin-6 (IL-6) pg/ml	<400	≥400		

High NUTRIC score 6–10 points				
Low NUTRIC score 0–5 points				
High modified NUTRIC score 5–9 points				
Low modified NUTRIC score 0–4 points				

APACHE II, Acute Physiology and Chronic Health Evaluation II; SOFA, Sequential Organ Function Assessment; ICU, Intensive Care Unit.

**Table 2:** The demographic and clinical characteristics of the patients.

	*n* (%)
Gender	
Male	200 (62.1)
Female	122 (37.9)

Comorbidities	
Diabetes mellitus	127 (39.4)
Hypertension	203 (63)
Coronary artery disease	94 (29.2)
Heart failure	24 (7.5)
Arrhythmia	18 (5.6)
Chronic obstructive pulmonary disease	40 (12.4)
Asthma	19 (5.9)
Kidney failure	29 (9)
Malignancy	24 (7.5)
History of cerebrovascular event	23 (7.1)
Alzheimer's disease	31 (9.6)
Parkinson's disease	3 (0.9)
Liver disease	4 (1.2)
Anxiety/panic disorder	9 (2.8)

Number of comorbidities	
0	49 (15.2)
1	50 (15.5)
2	101 (31.4)
3	83 (25.8)
4	28 (8.7)
5	11 (3.4)

Patient outcome	
Surviving	137 (42.5)
Exitus	185 (57.5)

Severity according to CDC	
Severe pneumonia	255 (79.2)
Critical disease	67 (20.8)
Nasal cannula/mask requirement	281 (87.3)
High-flow nasal oxygen requirement	223 (69.3)
Noninvasive mechanical ventilator requirement	73 (22.7)
Invasive mechanical ventilator requirement	189 (58.7)
Use of vasopressors on admittance to ICU	67 (20.8)
Requirement for vasopressors in ICU	145 (45)
Acute kidney injury	105 (32.6)
Requirement for dialysis and/or CRRT	71 (22)

CDC, Chinese Centre for Disease Control and Prevention; CRRT, Continuous Renal Replacement Therapy; ICU, Intensive Care Unit.

**Table 3:** Clinical and Laboratory test results.

	Mean ± SD
Age	69.1 ± 14.4
Number of days of initial symptoms in ICU	6.4 ± 3.4
Length of stay in ICU (days)	10.6 ± 7.0
Length of stay in hospital before admittance to ICU (days)	2.6 ± 3.2
Total length of hospital stay (days)	16.2 ± 10.5
Glasgow Coma Scale	13.1 ± 3.3
APACHE II	12.8 ± 7.1
SOFA score	4.4 ± 3.4
NUTRIC score	3.8 ± 2.0
Modified NUTRIC score	3.8 ± 1.9
Creatinine, mg/dL	1.40 ± 1.5
Albumin, g/dL	3.38 ± 0.5
Total bilirubin, *µ*mol/L	0.76 ± 10
D-Dimer, *µ*g/mL	5.2 ± 9.6
Procalcitonin, ng/ml	4.6 ± 32.2
Ferritin, ng/mL	1074.8 ± 1942.1
WBC, 10³/*µ*L	10.3 ± 5.7
Neutrophils, 10³/*µ*L	10.1 ± 25.4
Lymphocytes, 10³/*µ*L	1.07 ± 2.5
Haemoglobin, g/dL	12.7 ± 2.2
Thrombocytes, 10³/*µ*L	267.9 ± 120.0
Neutrophil/lymphocyte ratio	15.7 ± 16.7
Lactate, mmol/L	2.41 ± 1.2
IL-6, pg/ml	142.3 ± 249.4
CRP, mg/L	130 ± 160.4

APACHE II, Acute Physiology and Chronic Health Evaluation II; SOFA, Sequential Organ Function Assessment; WBC, white blood cell; CRP: C-reactive protein; IL-6, interleukin-6; ICU, Intensive Care Unit.

**Table 4:** Comparisons of the patient data in respect of the NUTRIC and mNUTRIC scores.

	NUTRIC score			Modified NUTRIC score		
	Low *n* = 260	High *n* = 62	*p* value	Low *n* = 236	High *n* = 86	*p* value
Age (years) (min–max)	67 (19–92)	80 (40–99)	0.0001	66 (19–92)	80 (36–99)	0.0001

Gender						
Male, *n* (%)	164 (63.1)	36 (58.1)	0.470	150 (63.6)	50 (58.1)	0.436
Female, *n* (%)	96 (36.9)	26 (41.9)		86 (36.4)	36 (41.9)	

Comorbidities						
Diabetes mellitus, *n* (%)	107 (41.2)	20 (32.3)	0.247	98 (41.5)	29 (33.7)	0.246
Hypertension, *n* (%)	159 (61.2)	44 (71.0)	0.188	142 (60.2)	61 (70.9)	0.090
Coronary artery disease, *n* (%)	71 (27.3)	23 (37.1)	0.161	62 (26.3)	32 (37.2)	0.071
Heart failure, *n* (%)	15 (5.8)	9 (14.5)	0.029	12 (5.1)	12 (14.0)	0.014
Arrhythmia, *n* (%)	12 (4.6)	6 (9.7)	0.128	8 (3.4)	10 (11.6)	0.010
Chronic obstructive pulmonary disease, *n* (%)	30 (11.5)	10 (16.1)	0.390	24 (10.2)	16 (18.6)	0.055
Asthma, *n* (%)	16 (6.2)	3 (4.8)	1.000	14 (5.9)	5 (5.8)	1.000
Renal failure, *n* (%)	12 (4.6)	17 (27.4)	0.0001	9 (3.8)	20 (23.3)	0.0001
Malignancy, *n* (%)	17 (6.5)	7 (11.3)	0.278	15 (6.4)	9 (10.5)	0.233
History of cerebrovascular event, *n* (%)	14 (5.4)	9 (14.5)	0.024	10 (4.2)	13 (15.1)	0.024
Alzheimer's disease, *n* (%)	21 (8.1)	10 (16.1)	0.089	16 (6.8)	15 (17.4)	0.009
Parkinson's disease, *n* (%)	2 (0.8)	1 (1.6)	0.475	1 (0.4)	2 (2.3)	0.175
Liver disease, *n* (%)	3 (1.2)	1 (1.6)	0.577	3 (1.3)	1 (1.2)	1.000
Anxiety/panic disorder, *n* (%)	6 (2.3)	3 (4.8)	0.382	5 (2.1)	4 (4.7)	0.255
Others, *n* (%)	17 (6.5)	2 (3.2)	0.547	17 (7.2)	2 (2.3)	0.115
Length of stay in ICU (days) (median) (min–max)	10 (2–41)	6 (2–43)	0.036	10 (2–41)	7 (2–43)	0.142
Length of stay in hospital before admittance to ICU (days) (median) (min–max)	2 (0–18)	2 (0–24)	0.091	2 (0–18)	2 (0–24)	0.148
Total length of hospital stay (days) (median) (min–max)	15 (2–69)	10 (2–46)	0.001	15 (2–69)	11 (2–46)	0.0001
Glasgow Coma Scale (median) (min–max)	15 (3–15)	7 (3–15)	0.0001	15 (6–15)	10 (3–15)	0.0001
Apache II (median) (min–max)	10 (2–34)	22 (3–39)	0.0001	9 (2–26)	21 (3–39)	0.0001
SOFA score (median) (min–max)	2 (1–9)	10 (4–16)	0.0001	2 (1–7)	9 (2–16)	0.0001

Patient outcome						
Survival, *n* (%)	136 (52.3)	1 (1.6)	0.0001	133 (56.4)	4 (4.7)	0.0001
Exitus, *n* (%)	124 (47.7)	61 (98.4)		103 (43.6)	82 (95.3)	

Severity according to CDC						
Severe pneumonia, *n* (%)	246 (94.6)	9 (14.5)	0.0001	227 (96.2)	28 (32.6)	0.0001
Critical disease, *n* (%)	14 (5.4)	53 (85.5)		9 (3.8)	58 (67.4)	
Nasal cannula/mask requirement, *n* (%)	255 (98.1)	26 (41.9)	0.0001	233 (98.7)	48 (55.8)	0.0001
High-flow nasal oxygen requirement, *n* (%)	199 (76.5)	24 (38.7)	0.0001	179 (75.8)	44 (51.2)	0.0001
Noninvasive mechanical ventilator requirement, *n* (%)	67 (25.8)	6 (9.7)	0.006	62 (26.3)	11 (12.8)	0.010
Invasive mechanical ventilator requirement, *n* (%)	127 (48.8)	62 (100.0)	0.0001	106 (44.9)	83 (96.5)	0.0001
Use of vasopressors on admittance to ICU, *n* (%)	14 (5.4)	53 (85.5)	0.0001	9 (3.8)	58 (67.4)	0.0001
Requirement for vasopressors in ICU, *n* (%)	85 (32.7)	60 (96.8)	0.0001	69 (29.2)	76 (88.4)	0.0001
Acute kidney injury, *n* (%)	58 (22.3)	47 (75.8)	0.0001	47 (19.9)	58 (67.4)	0.0001
Requirement for dialysis and/or CRRT, *n* (%)	38 (14.6)	33 (53.2)	0.0001	32 (13.6)	39 (45.3)	0.0001
Creatinine, mg/dL (median) (min–max)	0.89 (0.27–18.1)	2.22 (0.57–8.42)	0.0001	0.88 (0.27–7.41)	1.72 (0.47–18.1)	0.0001
Albumin, g/dL (median) (min–max)	3.5 (1.5–4.5)	3.2 (2.1–4.2)	0.0001	3.5 (1.5–4.5)	3.3 (2.1–4.2)	0.0001
Total bilirubin, *µ*mol/L (median) (min–max)	0.6 (0.1–2.7)	0.6 (0.1–17.6)	0.029	0.6 (0.1–2.7)	0.6 (0.1–17.6)	0.152
D-Dimer, *µ*g/mL (median) (min–max)	1.51 (0.19–80.0)	3.75 (0.6–35.2)	0.055	1.49 (0.19–70.9)	2.97 (0.2–80.0)	0.027
Procalcitonin, ng/ml (median (min–max)	0.19 (0.02–202.9)	0.95 (0.03–511.4)	0.001	0.18 (0.02–202.9)	0.68 (0.03–511.4)	0.009
Ferritin, ng/mL (median) (min–max)	597 (14–42079	867 (55–21309)	0.0001	600 (14–4207)	782 (55–21309)	0.0001
WBC, 10³/*µ*L (median) (min–max)	8.6 (1.53–26.8)	13.4 (0.12–39.5)	0.0001	8.67 (1.53–26.8)	11.3 (0.12–39.5)	0.0001
Neutrophils, 10³/*µ*L (median) (min–max)	7.2 (1.06–456)	11.6 (0.04–26.4)	0.621	7.23 (1.06–24.9)	9.91 (0.04–456)	0.016
Lymphocytes, 10³/*µ*L (median) (min–max)	0.65 (0.05–20.6)	0.61 (0.02–27.7)	0.031	0.66 (0.05–20.6)	0.61 (0.02–27.7)	0.146
Haemoglobin, g/dL (median) (min–max)	13.1 (6.1–17.2)	12.1 (5.8–16.9)	0.0001	13.2 (6.1–17.2)	12.1 (5.8–16.9)	0.0001
Thrombocytes, 10³/*µ*L (median) (min–max)	252 (34–767)	227 (23–613)	0.205	252 (64–767)	235 (23–613)	0.041
Neutrophil/Lymphocyte ratio (median) (min–max)	10.4 (0.14–165.2)	15.9 (0.13–70.6)	0.010	10.3 (0.14–165.2)	14.2 (0.13–70.6)	0.023
Lactate, mmol/L (median) (min–max)	2.07 (0.5–6.9)	2.38 (0.67–7.1)	0.031	2.08 (0.55–6.95)	2.31 (0.59–7.07)	0.113
IL-6, pg/ml (median) (min–max)	49 (2–1492)	124 (9.4–2150)	0.0001	48.9 (2.1–1492)	91.6 (2–2150)	0.001
CRP, mg/L (median) (min–max)	133 (1–335)	148 (1–373)	0.273	133 (1–335)	141 (1–373)	0.489

APACHE II, Acute Physiology and Chronic Health Evaluation II; SOFA, Sequential Organ Function Assessment; WBC: white blood cell; CRP, C-reactive protein; IL-6, interleukin-6; ICU, Intensive Care Unit. *p* value < 0.05 is statistically significant.

**Table 5:** AUC values of the scores.

	AUC	95% CI AUC				
		Lower bound	Upper bound	Cutoff value	Sensitivity %/specificity %	*p* value
NUTRIC score	0.791	0.743	0.840	>3.5	68.6/79.1	0.0001
Modified NUTRIC score	0.786	0.737	0.835	>3.5	67.7/80.0	0.0001
Glasgow Coma Scale	0.791	0.742	0.841	<14.5	92.0/65.9	0.0001
APACHE II	0.880	0.845	0.916	>10.5	77.3/78.1	0.0001
SOFA score	0.829	0.785	0.874	>2.5	82.2/73.7	0.0001

AUC, area under the curve; CI, confidence interval; APACHE II, Acute Physiology and Chronic Health Evaluation II; SOFA, Sequential Organ Function Assessment. *p* value < 0.05 is statistically significant.

## Data Availability

The data that support the findings are available from the corresponding author (Dr Berkay Kucuk) upon reasonable request.

## Additional Points

*What Is Known?* (i) The NUTRIC score is calculated by evaluating age, APACHE II score, SOFA score, number of comorbidities, length of hospital stay before ICU, and serum interleukin-6 (IL-6) level. (ii) If the serum interleukin-6 (IL-6) level is not evaluated from these parameters, it is called the modified NUTRIC score. (iii) The NUTRIC score and the modified NUTRIC score are two scoring systems that show the nutritional risk status and severity of acute disease of patients. *What Is New?* (i) This study shows that the NUTRIC score and the modified NUTRIC score are predictors of mortality in COVID-19 intensive care patients. (ii) When IL-6 cannot be examined, the modified NUTRIC score can be considered safe and effective for the prediction of mortality in COVID-19 patients.
